# Editorial: Arbuscular mycorrhiza-mediated augmentation of plant secondary metabolite production

**DOI:** 10.3389/fpls.2023.1150900

**Published:** 2023-02-13

**Authors:** Qiang-Sheng Wu, Fábio S. B. Silva, Mohamed Hijri, Rupam Kapoor

**Affiliations:** ^1^ College of Horticulture and Gardening, Yangtze University, Jingzhou, China; ^2^ Institute of Biological Sciences, University of Pernambuco, Recife, Brazil; ^3^ Institut de Recherche en Biologie Végétale, Département de Sciences Biologiques, Université de Montréal, Montréal, QC, Canada; ^4^ African Genome Center, Mohammed VI Polytechnic University, Ben Guerir, Morocco; ^5^ Department of Botany, Faculty of Science, University of Delhi, Delhi, India

**Keywords:** metabolome, nutrient acquisition, plant metabolism, biotic stress, symbiotic interaction

The health promoting properties of plant products have increasingly gained acceptance. Besides several factors such as plant genotype, cultivation practices, environmental conditions, abiotic and biotic stress, symbiotic association of plant roots with soil-dwelling fungi namely arbuscular mycorrhizal (AM) fungi (AMF) is known to influence the content and range of phytochemicals produced by plants (Sbrana et al., 2014; Kapoor et al., 2017; Fokom et al., 2019; Thokchom et al., 2020; Javanmard et al., 2022; Zhao et al., 2022). The literature is overwhelmed with studies that deliberate on the effect of AMF on plants’ metabolism, however knowledge on the underlying mechanisms thereof remains fragmentary. The four articles hosted in this special issue consolidate our understanding on the wide range of effects of AMF on plant metabolism and implication it holds on the overall growth and performance of the plant.

Although AMF are restricted morphologically to the roots, AMF-induced physiological and metabolic alterations in the root also influence the physiology of the entire plant (Schweiger and Muller, 2015). Since the fungal symbionts are dependent upon plant-derived photosynthates (carbohydrates) and fatty acids, they act as strong carbon sink in roots. Consequently, the carbon balance in plants is maintained by regulation of photosynthesis and leaf primary metabolism (Kaschuk et al., 2009; Kogel et al., 2010). Alteration in the secondary metabolite profile of plant is an inevitable consequence of the changes in primary metabolism. While AMF-mediated effects on plant primary metabolism have been a subject of landscape of studies, relatively little is known vis-à-vis modifications in secondary metabolism in the systemic tissues. Furthermore, a comparative analysis of diverse plant organs in terms of metabolic traits, biomass/allocation patterns, and transcript profiles would assist in comprehensive understanding of plant’s response to AMF inoculation. In this direction, to ascertain the consequences of AMF inoculation on enhanced root development and shoot growth in apple plants, Jing et al. mapped the metabolic pathways on transcriptome and metabolome data. They observed the involvement of multiple pathways in promotion of root development in mycorrhized plants and reported that sugar, fatty acid and organic acid metabolisms in roots could be regulated by arbuscular mycorrhizal (AM) formation. Furthermore, the alteration in hormonal levels also coordinated with the expression levels of different genes associated with hormone synthesis. The ratio of auxin to cytokinin increased following formation of AM that favored root development. Although root-to-shoot ratios in terms of growth were same in inoculated and non-inoculated plants, several metabolites specifically accumulated in the shoot, out of which some were exclusively present either in non-mycorrhizal or mycorrhizal plants. The morphology of shoot cells also changed in mycorrhizal plants, which was related to AM-mediated effect on the transcription of morphogenesis-related genes.

A large proportion of reports on the effect of AMF on plant growth and development are based on studies conducted under controlled environment using sterile soil that eliminates interference of other factors (Shtark et al., 2021; Fayuan et al., 2022; Qi et al., 2022). However, under natural conditions, when inoculated in soil, AMF interact with the indigenous microorganisms and their efficacy to improve plant growth and nutrient uptake is significantly influenced (Marulanda-Aguirre et al., 2008; Nacoon et al., 2020; Sangwan and Prasanna, 2022). The molecular mechanisms by which the indigenous microorganisms regulate AM functions are obscure. Ren et al. showed that indigenous microorganisms counteract the beneficial effects of AMF on the adaptability of a pioneer species *Bidens tripartita* to establish itself in a fragile terrestrial ecosystem - Karst that is characterized by nutrient deficiency. Indigenous microorganisms downregulated the AM-induced genes related to P and N metabolism. The study emphasizes the need to consider interactions of AM with native microorganisms before introducing them into an ecosystem.

AMF have been reported to increase plant tolerance to both biotic and abiotic stresses, and congruently secondary metabolites facilitate plants to endure many abiotic and biotic stress conditions (Korenblum and Aharoni, 2019; Begum et al., 2021; Sarkar and Sadhukhan, 2023). However, influence of AMF inoculation in concurrence with abiotic/biotic stress on fluxes in secondary metabolism has received relatively less consideration (Korenblum and Aharoni, 2019; Begum et al., 2021). Under state of nutrient inadequacy, relationship between plant growth and secondary metabolism and reciprocity in C distribution among functional C resources have been a matter of interest (Hartmann et al., 2020; Xie et al., 2022). Xie et al. explored the effect of interaction between plant growth stage and AM symbiosis on C partitioning among various resources. They demonstrated an important role of AM symbiosis in upholding plant growth under nutrient constrain, from the viewpoint of C partitioning. Studies of this kind will legislate ground for impending studies to investigate principal mechanisms of AM symbiosis in plant secondary metabolism, especially under nutrient stress and particularly in field conditions, to obtain a viewpoint on overall functionality of symbiosis. Such studies can also be outstretched to other stresses of biotic or abiotic origin, and the pattern of trade-offs and their specificity towards AMF strains, type and intensity of stress, and developmental stage of the plant can be examined.

Regarding herbivory, it is well known that plants have evolved a suite of defense mechanisms, one of which is association with AMF, which results in quick and robust stress responses to notorious herbivores. Du et al. provides a significant piece of evidence in this direction by showing that amendment of *Ageratina adenophor* with AM fungi could facilitate its invasiveness by inducing chemical defense, thereby making the weed more tolerant to herbivory by *Aphis gossypii*. The colonization by the two dominant AMF species i.e., *Claroideoglomus etunicatum* and *Septoglomus constrictum* decreased the feeding of the generalist herbivore *A. gosypii* by increasing the levels of flavonoids and phenols in the plant. The study also suggested ecological implications of AMF community in management of invasive weeds.

To summarize, this article assembles studies of interactions between AMF and plants in metabolic context, and thus enables valuable insights into designing better experimental approach so as to understand the diverse roles of AMF in maintenance of plant health and production within the low-input, sustainable cropping systems.

## Author contributions

RK wrote the editorial and all the authors have collectively reviewed and approved the submitted version.
